# Effects of acetaminophen ingestion on the torque–duration relationship and fatigability during intermittent exercise in patients with type 1 diabetes

**DOI:** 10.1113/EP093694

**Published:** 2026-10-27

**Authors:** Nadia Fekih, Amal Machfer, Achraf Ammar, Firas Zghal, Mohamed Amine Bouzid

**Affiliations:** ^1^ Research Laboratory: Education, Motricité, Sport et Santé, EM2S, LR19JS01, High Institute of Sport and Physical Education University of Sfax Sfax Tunisia; ^2^ Department of Training and Movement Science Institute of Sport Science Johannes Gutenberg‐University Mainz Mainz Germany; ^3^ Department of Nutrition and Food Technology School of Agriculture The University of Jordan Amman Jordan; ^4^ High Institute of Sport and Physical Education of Sfax University of Sfax Sfax Tunisia; ^5^ Research Laboratory Molecular Bases of Human Pathology LR19ES13, Faculty of Medicine University of Sfax Sfax Tunisia; ^6^ CIAMS Université Paris‐Saclay Orsay France

**Keywords:** acetaminophen, critical torque, neuromuscular fatigue, type 1 diabetes, Pan African Clinical Trial Registry Identifier: PACTR202510823123375. Registered trial name: Neuromuscular fatigue regulation and metabolic disorders during exercise in patients with type 1 diabetes

## Abstract

This study aimed to determine the effects of acute ingestion of acetaminophen (ACT) on the magnitude of exercise‐induced peripheral fatigue (ΔPtw) in patients with type 1 diabetes (T1D). In a randomized, placebo‐controlled, double‐blind design, 22 males (11 T1D and 11 control (CTR) completed 60 maximum voluntary contractions 60 min after ingesting 1 g of acetaminophen (ACT) or 1 g of placebo (PL) on two separate visits. Central and peripheral fatigue were assessed from pre‐ to post‐exercise changes in quadriceps voluntary activation (ΔVA) and potentiated twitch force (ΔPtw), respectively. Critical torque (CT) was defined as the mean force produced during the final 12 contractions, and work performed (*W*′) was computed as the impulse performed above CT. Maximal voluntary contraction (MVC), VA and Ptw decreased after exercise in both groups, with no significant difference between PL and ACT condition. Compared to PL, ACT increased root mean square/*M*_max_ and CT values in the T1D (+21% and 8% respectively) and CTR groups (+40% and +19%; respectively), with significant lower effect in the T1D compared to the CTR group (*P *< 0.05). No significant effect of ACT was observed for *W*′ in the T1D (+7%) and CTR groups (+4%) (*P* = 0.68). These findings indicate that acute ACT ingestion increased CT and muscle activation during the repeated‐MVC protocol in both groups, although the magnitude of these responses was smaller in the T1D than the CTR group.

## Abstract (highlights)

- **What is the central question of this study?**
  Does acute acetaminophen (1 g) alter exercise‐induced quadriceps neuromuscular fatigue and the force–duration parameters (critical torque (CT), work performed (*W*′)) in type 1 diabetes (T1D) patients versus matched controls?
- **What is the main finding and its importance?**
  Acetaminophen increased muscle activation and CT in both groups, although these responses were smaller in T1D, whereas *W*′ and the magnitude of peripheral fatigue, quantified using ΔPtw, were unchanged. These hypothesis‐generating findings suggest a blunted neuromuscular response to acetaminophen in men with T1D and support further investigation of acetaminophen as a potential experimental probe of neuromuscular responsiveness rather than as a clinical detection or diagnostic method.

## 1 INTRODUCTION

Although exercise and physical activity are well‐established strategies for reducing cardiovascular risk, emerging evidence indicates that individuals with type 1 diabetes (T1D) may present greater fatigability and reduced muscle strength. For instance, T1D has been linked to lower muscle fibre volume and diminished maximal isometric force production (Krause et al., 2011). In addition, patients with T1D exhibit slower motor unit discharge rates, reflecting reduced motor neuron drive to the muscle, and reach task failure ∼45% sooner than healthy controls during sustained submaximal knee extensor contractions, suggesting a meaningful contribution of central fatigue (Almeida et al., 2008). However, the mechanisms underlying this fatigability during exercise in the T1D population are not well understood.

Skeletal muscle fatigability is a reversible, short‐term, activity‐induced decline in muscle strength or power (Gandevia, 2001) that can impair the ability to perform everyday activities requiring repeated or sustained contractions (Enoka & Duchateau, 2008). This temporary reduction in force‐generating capacity arises from central and/or peripheral mechanisms. Central fatigue refers to a reduction in voluntary neural drive arising proximal to the neuromuscular junction, including supraspinal, spinal and peripheral nerve processes involved in muscle activation, whereas peripheral fatigue refers to mechanisms distal to the neuromuscular junction that reduce the muscle's force‐generating capacity (Allen et al., 2008).

Peripheral fatigue has been proposed to be regulated to avoid exceeding an individual, task‐specific ‘critical’ threshold during exercise (Hureau et al., 2018). Feedback from group III/IV muscle afferents to the central nervous system (CNS) may modulate central motor drive by reducing motoneuronal output during fatiguing exercise and thereby limit the development of peripheral fatigue (Amann et al., 2009; Blain et al., 2016; Hureau et al., 2019). There is considerable evidence of a critical threshold of peripheral fatigue in healthy subjects (Amann & Calbet, 2008; Burnley, 2009; Hureau et al., 2014), elderly (Zarzissi et al., 2020) and in people with T1D (Fekih et al., 2024). Critical torque (CT) should not be interpreted as synonymous with a critical peripheral fatigue threshold. Rather, CT represents an experimentally derived threshold of the torque–duration relationship, whereas the notion that exercise tolerance is regulated by the attainment of a specific level of peripheral fatigue remains a proposed physiological explanation supported by indirect evidence.

Studies suggest that the effects of T1D on group III/IV muscle afferents evolve over the course of the disease (Sveen et al., 2013; Grotle et al., 2017). However, this evidence was derived from adults with long‐standing T1D evaluated for small‐ and large‐fibre neuropathy and from a rodent model of T1D. These findings should therefore be extrapolated cautiously to young adults with T1D without clinically significant neuropathy. Given the purported influence of group III/IV muscle afferents on exercise performance (Broxterman et al., 2015; Hureau et al., 2019), research has shown that attenuation of these afferents by intrathecal fentanyl leads to increased peripheral fatigue (Amann & Calbet, 2008; Amann et al., 2009; Blain et al., 2016). Therefore, several pieces of scientific evidence have shown that the level of neuromuscular activation and the development of neuromuscular fatigue can be modulated by therapeutic interventions that have the potential to reduce the sensation of muscle pain by acting at the level of the central nervous system (Morgan, Bowtell et al., 2018). One such intervention is the use of acetaminophen (ACT; or paracetamol).

ACT is an analgesic drug that is not prohibited under current anti‐doping regulation (Morgan et al., 2018; Toussaint et al., 2010). ACT is reported to improve endurance performance, muscle activation and critical torque during isometric exercise (Morgan et al., 2018) and also during endurance exercise (Mauger et al., 2010). Although the mechanisms of its analgesic effects are not yet fully understood, ACT appears to act predominantly within the central components of the nervous system rather than on the peripheral components (Graham et al., 2013; Toussaint et al., 2010). Cyclooxygenase inhibition may reduce prostaglandin synthesis and attenuate group III/IV muscle‐afferent discharge Indeed, it has been shown that inhibition of cyclooxygenase, the enzyme responsible for synthesizing prostaglandin from arachidonic acid, is the main action of ACT (Anderson, 2008). Given its role in stimulating the discharge of III/IV afferents (Schaible et al., 2011), inhibiting prostaglandin synthesis could reduce the activity of these afferences (Hayes et al., 2006). As a result, the level of muscle activation will be maintained and the CNS will keep stimulating active muscles (Amann & Calbet, 2008). These mechanisms likely work in concert to reduce pain perception following acute ACT ingestion by raising the nociceptive input required to produce a given level of perceived pain. Therefore, coupling ACT ingestion with neuromuscular assessment would provide an idea of neuromuscular function during exercise, specifically III/IV muscle afferent activity. However, no study has investigated the neuromuscular basis of exercise‐induced fatigue following ACT ingestion in patients with T1D. Such data would provide new insights into reduced exercise capacity and exacerbated neuromuscular fatigue in this population.

Furthermore, ACT ingestion could modulate aspects of power or the power–duration relationship. CT represents the asymptote of the hyperbolic torque–duration relationship. In accordance with the method described by Burnley (2009), CT was defined as the mean force produced during the final 12 contractions of the 60 maximal voluntary contractions (MVCs) protocol, while work performed (*W*′) has been calculated as the area above CT (Burnley, 2009)

Together, CT (or the analogous critical power/critical velocity in other exercise modalities) and *W*′ (or its equivalent) can be used to predict exercise performance with good accuracy (Burnley et al., 2012). From a physiological perspective, CT reflects the highest oxidative metabolic rate that can be sustained without a progressive depletion of *W*′ (Poole et al., 1988). The extent of *W*′ depletion is closely linked to the development of neuromuscular fatigue (Zarzissi et al., 2020) and reductions in muscular efficiency (Murgatroyd & Wylde, 2011). Morgan et al. (2018) reported that acute ACT ingestion increased CT and was accompanied by greater muscle activation during the later stages of a 60‐MVC protocol in healthy individuals (Morgan et al., 2018). Collectively, these findings suggest that ACT may attenuate fatigue during repeated maximal contractions by preserving muscle activation. Accordingly, it is of interest to examine the effects of ACT ingestion on CT and *W*′ in patients with T1D, to provide additional bioenergetic insight into the mechanisms underpinning neuromuscular fatigue and exercise capacity in this population.

Therefore, the present study aimed to examine the effects of ACT ingestion on exercise‐induced quadriceps neuromuscular fatigue and to characterize the force–duration relationship parameters in individuals with T1D compared with well‐matched healthy controls. It was hypothesized that ACT ingestion would increase CT and improve force maintenance during the repeated contraction protocol in both groups. Given the altered neuromuscular function previously reported in T1D, we further explored whether the magnitude of this response differed between groups. In the present study, peripheral fatigue was quantified as the reduction in potentiated twitch force from baseline to post‐exercise (ΔPtw), which represents the magnitude of exercise‐induced contractile impairment. CT is a distinct force–duration parameter and was estimated as the mean torque produced during the final 12 contractions, whereas *W*′ represents the finite impulse accumulated above CT. The term ‘critical peripheral fatigue threshold’ is used only to refer to the theoretical proposition that exercise performance may be regulated around a task‐specific limit of peripheral fatigue. Accordingly, neither CT nor ΔPtw should be considered a direct or interchangeable measure of this theoretical construct.

## 2 METHODS

### 2.1 Participants

Twenty‐two sedentary males (11 T1D, 23 ± 4 years old; 11 healthy controls (CTR)), 23 ± 3 years old) volunteered to take part in the study. To be eligible, participants with T1D were required to meet the following criteria: aged 18–35 years, diagnosed with T1D for at least 3 years, and having glycated haemoglobin (HbA1c) <10% over the 6 months prior to enrolment. Exclusion criteria included clinically significant diabetes‐related complications that could limit exercise participation (e.g., diabetic foot, retinopathy or severe neuropathy), uncontrolled hypertension, a recent episode of diabetic ketoacidosis, severe hypoglycaemia within the previous 3 months, and current use of lipid‐lowering therapy. All control participants had no symptoms of cardiac or metabolic disease and no contraindications to physical exercise. Participants with T1D and healthy subjects were matched for anthropometric measurements (Table 1). All participants completed a medical health questionnaire to confirm that they could safely consume ACT before undertaking the exercise protocols. The questionnaire included items on medication allergies, current medication use, prior ACT consumption and relevant medical history.

**TABLE 1:** Participant characteristics

	T1D group (*n* = 11)	CTR group (*n* = 11)	*P*
Age (years)	23.3 ± 4.1	22.8 ± 3.2	NS
Fat mass (%)	16.7 ± 2.7	15.9 ± 1.8	NS
Height (m)	1.7 ± 0.1	1.8 ± 0.1	NS
Body mass (kg)	68.4 ± 4.3	71.2 ± 5.4	NS
BMI (kg m^−2^)	22.9 ± 2.4	22.4 ± 1.4	NS
HbA_1c_ (%)	8.5 ± 0.6	5.5 ± 0.4	<0.05
Diabetes duration (years)	15.6 ± 5.6	—	
Total insulin dose per day (U kg^−1^)	0.6 ± 0.1	—	
Physical activity			
Leisure activity per MAQ (MET‐h week^−1^)	15.6 ± 8.2	17.1 ± 9.5	NS
Total activity per MAQ (MET‐h week^−1^)	53.6 ± 11.2	54.4 ± 10.5	NS

Abbreviations: BMI, body mass index; HbA1c, glycated haemoglobin; MAQ, Modifiable Activity Questionnaire; MET, metabolic equivalent of task.

The study was approved by the Regional Research Ethics Committee (CPP SUD No. 2021/0427) and conducted in accordance with the *Declaration of Helsinki*. Participants were fully informed of the procedure and the risks involved and gave written consent.

### 2.2 Experimental protocol

This study utilized a randomized, placebo‐controlled, double‐blind design. Participants in the T1D and control groups attended the laboratory on three occasions separated by 3–5 days: one familiarization visit and two experimental visits during which the fatiguing exercise protocol was completed. Prior to each visit, participants were instructed to abstain from caffeine for 12 h, and from strenuous exercise and alcohol for 24 h. They were also asked to avoid analgesics and any anti‐inflammatory medication throughout the study and to arrive well‐rested and adequately hydrated.

#### 2.2.1 Admission visit

To ensure a stable level of performance, participants attended a familiarization session 4 days before the experimental trials, during which they were introduced to all procedures. Upon arrival at the laboratory, anthropometric measurements were obtained, and participants were familiarized with producing and sustaining maximal effort on an isometric dynamometer. Leg dominance was also assessed during this visit.

#### 2.2.2 Interventional visits

During visits 2 and 3, participants performed the fatiguing exercise protocol (60 isometric maximal voluntary contractions) following the ingestion of either capsule of ACT (1 g) or a placebo control (PL) (1 g). PL and ACT were administered as two identically coloured capsules. The placebo consisted of maltodextrin powder enclosed in gelatin capsules, matched in appearance to ACT but without analgesic or antipyretic effects. Capsules were ingested 60 min before exercise to coincide with the approximate time of peak plasma ACT concentration, which typically occurs ∼60 min post‐ingestion (Anderson, 2008; Forrest et al., 1982)

Visits 2 and 3 were performed in a double‐blind, randomized design. In each session participants were asked to perform 60 isometric MVCs over a 5‐min period (3 s of contraction, 2 s of relaxation) with the knee extensors of the dominant leg (Burnley, 2009). A visual feedback of force output was provided on the computer screen to maximize performance. Neuromuscular function (MVC, quadriceps muscle activation: EMG; voluntary activation: VA and potentiated twitch: Ptw) has been evaluated before and directly after the fatiguing exercise protocol. For each participant, both sessions were conducted at the same time of day and under identical ambient conditions (25 ± 1°C).

### 2.3 Data collection and analysis

#### 2.3.1 Neuromuscular function assessment of the quadriceps

Participants were seated on an isometric dynamometer (Good Strength, Metitur Oy, Jyväskylä, Finland) equipped with a cuff connected to a strain gauge. The cuff was secured ∼2 cm above the lateral malleolus of the dominant leg using a non‐compliant Velcro strap to allow continuous recording of quadriceps force. The knee joint was positioned at 90° of flexion (0° = full extension).

Femoral nerve stimulation was delivered percutaneously using a constant‐current stimulator (Digitimer Ltd, Welwyn Garden City, UK) producing single square‐wave pulses (1 ms; maximal voltage 400 V). The cathode (10‐mm diameter Ag–AgCl self‐adhesive electrode) was placed over the femoral nerve within the femoral triangle, and the anode (10 × 5 cm self‐adhesive electrode; Compex Medical SA, Ecublens, Switzerland) was positioned midway between the greater trochanter and the iliac crest.

Optimal stimulation intensity was established before each session from M‐wave and force responses. Current was increased in 5 mA increments until a plateau was reached in evoked knee‐extensor force and in the peak‐to‐peak maximal M‐wave amplitude (*M*_max_) recorded from the vastus lateralis (VL), vastus medialis (VM) and rectus femoris (RF). The stimulation intensity used for testing was then set at 150% of this value (supramaximal) to minimize the potential influence of axonal hyperpolarization (Burke, 2002).

During each MVC, two stimuli were delivered to the femoral nerve: one during the MVC (superimposed twitch) and a second 3 s after the MVC (potentiated twitch, Ptw). This delay allows a potentiated response, improving measurement reliability compared with non‐potentiated twitches (Kufel et al., 2002). The magnitude of exercise‐induced peripheral fatigue was quantified as the change in Ptw from baseline to post‐exercise (ΔPtw), while global fatigue was calculated as the change in MVC from baseline to post‐exercise (ΔMVC). Central fatigue was assessed via voluntary activation (VA) using the twitch interpolation technique as follows (Merton, 1954):

$$\mathrm{Voluntary}\;\mathrm{activation}\;\%\;=\left[1\;-\;\left(\mathrm{SIT}/\mathrm{Ptw}\right)\right]\;\times \;100$$

where SIT represents the amplitude of the superimposed twitch evoked during the plateau phase of the MVC, and Ptw represents the amplitude of the potentiated twitch elicited 3 s after the contraction. SIT and Ptw amplitudes were determined from the peak force response of the evoked twitch. The superimposed twitch was expressed relative to the potentiated twitch amplitude, and no additional torque correction factor was applied because stimulation occurred during the plateau of maximal voluntary torque.

#### 2.3.2 Electromyography

Bipolar silver chloride surface electrodes (Dormo Electrodes, SX‐50 ECG, Telic, S.A.U., Spain) were used to record electromyographic (EMG) activity. Electrodes were aligned longitudinally over the muscle bellies in accordance with SENIAM recommendations, with a 20‐mm inter‐electrode distance, and a reference electrode was placed over the patella. Skin preparation (shaving, light abrasion and cleaning with alcohol) was performed to achieve low impedance at the electrode–skin interface (*Z* < 5 kΩ). Electrode positions were marked with indelible ink to ensure consistent placement across visits.

Signals were amplified (Octal Bio Amp ML 138, ADInstruments, Bella Vista, Australia; bandwidth 10 Hz to 1 kHz, common‐mode rejection ratio >96 dB, gain = 1000) and digitized simultaneously with force data using an acquisition system (Powerlab 16SP, ADInstruments) and LabChart 7.0 software (ADInstruments) at a sampling frequency of 2 kHz. For each potentiated twitch (Ptw), the peak‐to‐peak amplitude of the maximal compound muscle action potential (M‐wave; *M*_max_) was determined. During the 5‐min MVC protocol, EMG activity from the VL, VM and RF was quantified using the root mean square (RMS) and summed across muscles. RMS was calculated over a fixed 0.5‐s window immediately preceding the superimposed stimulation artefact, corresponding to the plateau phase of the MVC, with visual inspection used to confirm that the selected window did not contain stimulation artefacts or signal disturbances. RMS values were normalized to the maximal compound muscle action potential (*M*_max_) obtained during the corresponding contraction (RMS/*M*_max_) in order to account for potential changes in peripheral excitability during the exercise protocol. The normalized RMS values from VL, VM and RF were summed to provide an index of global quadriceps muscle activation

#### 2.3.3 Determination of CT and *W*′

During the 60‐MVC test, CT was defined as the mean force produced during the final 12 contractions (i.e., the last 60 s). No additional slope‐ or variance‐based stabilization criterion was applied. *W*′ was calculated as the impulse above CT, corresponding to the area above the CT threshold on the force–time curve during the exercise (Burnley, 2009).

### 2.4 Statistical analyses

The sample size was estimated from a previous study reporting MVC differences between individuals with T1D and healthy controls (Almeida et al., 2008). Assuming an effect size of 0.93, α = 0.05 and β = 0.80, a minimum of 10 participants per group was required to detect a between‐group difference in maximal voluntary force using a two‐way repeated‐measures ANOVA (G*Power, version 3.1.9.4) (Faul et al., 2007).

Data are presented as means ± SD and were analysed using Statistica for Windows (version 12.0, TIBCO Software, Palo Alto, CA). Normality and homogeneity of variance were verified using the Shapiro–Wilk and Levene tests, respectively. CT and *W*′ were compared using a two‐way ANOVA (group × condition). For MVC, VA, Ptw, RMS/*M*_max_ and M‐wave variables, a repeated‐measures ANOVA (group × condition × time) was conducted. When a significant main effect or interaction was detected, Bonferroni‐adjusted *post hoc* pairwise comparisons were performed within that specific outcome variable to control for multiple comparisons among the relevant factor levels. No *post hoc* comparisons were conducted in the absence of a significant ANOVA effect. Statistical significance was set at *P* < 0.05.

## 3 RESULTS

### 3.1 Baseline neuromuscular function

Neuromuscular function at baseline (MVC, Ptw and *M*_max_) was similar between PL and ACT conditions for both groups (Table 2). However, although there was a similar VA level between groups (*P* = 0.23), baseline MVC, Ptw and *M*_max_ values were lower in the T1D group compared to the CTR group (*P* < 0.05).

**TABLE 2:** Effect of repeated MVCs on quadriceps neuromuscular function

	CTR				T1D				Effects
	PL		ACT		PL		ACT		
	Pre	Post	Pre	Post	Pre	Post	Pre	Post	
**MVC (N)**	632.9 ± 94.8	468.4 ± 40.1^*^	684.3 ± 106.5	485.6 ± 52.3^*^	522.1 ± 131.2^**^	351 ± 97.8^*^	540.7 ± 116.1^**^	372.8 ± 56.3^*^	Interaction *P* < 0.001 Group *P* < 0.001 Condition *P* = 0.45 Time *P* < 0.001
**Ptw (N)**	162.6 ± 23.1	79.3 ± 15.8^*^	164.1 ± 14.3	73 ± 27^*^	138.8 ± 21.3^**^	80.9 ± 29.3^*^	140.9 ± 18.1^**^	79 ± 22^*^	Interaction *P* < 0.01 Group *P* = 0.03 Condition *P* = 0.77 Time *P* < 0.01
**VA (%)**	95.1 ± 1.5	89.8 ± 1.6^*^	96.4 ± 1.3	89.3 ± 7.6^*^	93 ± 3	86.5 ± 4.1^*^	94 ± 2.7	89.8 ± 4.8^*^	Interaction *P* = 0.02 Group *P* = 0.03 Condition *P* = 0.45 Time *P* < 0.001
**VL *M*_max_ (mV)**	7.2 ± 3.7	4.6 ± 1.8^*^	6.4 ± 3.3	5.1 ± 2.9^*^	5.2 ± 2^**^	3.5 ± 1.7^*^	5.2 ± 1.2	4.6 ± 1.3	Interaction *P* < 0.01 Group *P* < 0.01 Condition *P* = 0.68 Time *P* = 0.04
**VM *M*_max_ (mV)**	7.6 ± 3.5	5.2 ± 3.1^*^	6.8 ± 3.4	5.8 ± 3.3	6.1 ± 2.1^**^	4.4 ± 2.2^*^	5.3 ± 2.7^**^	4.1 ± 2.5	Interaction *P* < 0.01 Group *P* < 0.01 Condition *P* = 0.32 Time *P* = 0.04
**RF *M*_max_ (mV)**	5.5 ± 3.4	4.3 ± 3.3^*^	5.8 ± 1.6	4.5 ± 2^*^	5.1 ± 2.4	3.9 ± 1.7^*^	5.4 ± 2	3.9 ± 1.4^*^	Interaction *P* < 0.001 Group *P* = 0.04 Condition *P* = 0.24 Time *P* < 0.001

^∗^ *P* < 0.05 versus Baseline values;

** *P* < 0.05 versus CTR values.

Abbreviations: ACT, paracetamol; MVC, maximal isometric voluntary contraction; *M*_max_, maximal muscle action potential; PL, placebo; Ptw quadriceps potentiated twitch force; RF, rectus femoris; VA, voluntary activation of quadriceps motor units; VL, vastus lateralis; VM, vastus medialis.

### 3.2 60‐MVCs protocol

#### 3.2.1 Exercise performance

The evolution of force (% MVC from baseline) for both groups during the exercise is presented in Figure 1a,b. In both conditions (ACT and PL), MVC values decreased over time and stabilized in the last 12 contractions for both groups (T1D and CTR). The decrease in force was similar between ACT and PL condition from the first contraction to the 27th contraction in both conditions for the T1D group (*P* = 0.44) and up to the 23rd contraction for the CTR group (*P* = 0.53). Thereafter, MVC values decreased at a slower rate in the ACT condition compared with the PL condition in the T1D (mean improvement: +9%) and CTR group (mean improvement: +9.5%) until the last contraction of the exercise (*P *< 0.05).

**FIGURE 1:**
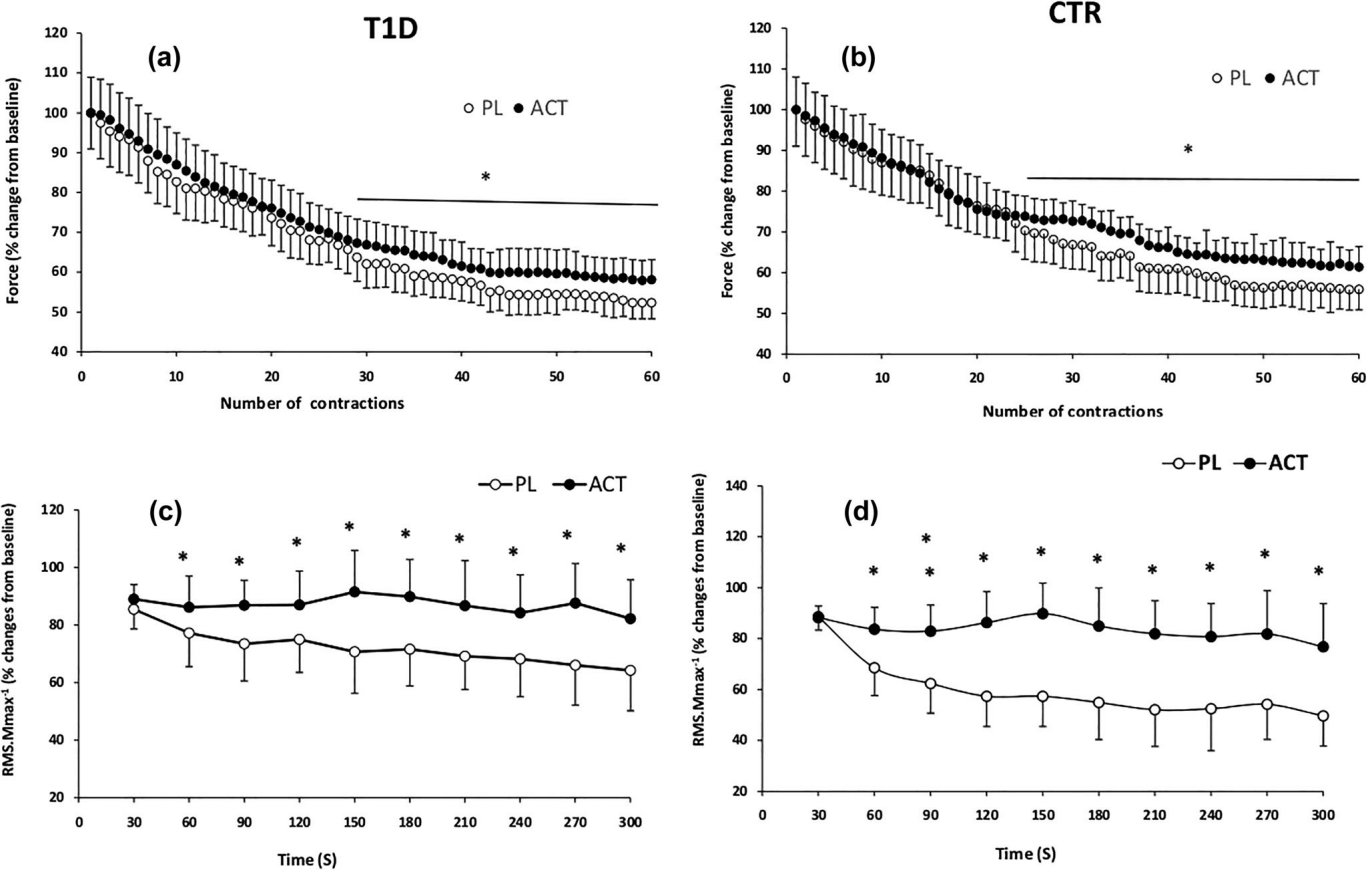
(a, b) Maximal voluntary contractions (MVC) during the 60 maximal contractions with (ACT) and without (PL) acetaminophen in control (CTR, b) and type 1 diabetes (T1D, a) participants. (c, d) Quadriceps muscle activation (RMS/*M*_max_) during the 60 maximal voluntary contractions with (ACT) and without (PL) acetaminophen in CTR (d) and T1D (c) participants. ^∗^ *P* < 0.05 versus PL.

#### 3.2.2 Electromyography

The evolution of RMS/*M*_max_ values (averages of six contractions (30 s)) during exercise is presented in Figure 1c,d. At the beginning of exercise (first 30 s of exercise), RMS/*M*_max_ was similar between the two conditions (ACT and PL) in the T1D group (89 ± 5% vs. 86 ± 7%, *P* = 0.16) and in the CTR group (88 ± 5% vs. 89 ± 5%, *P* = 0.87).

From the first minute to the end of exercise, RMS/*M*_max_ was improved in the ACT condition compared with the PL condition (mean improvement: 21 ± 7% in the T1D group vs. 40 ± 8% in the CTR group) (Figure 1c,d). However, this improvement was significantly greater in the CTR group compared with the T1D group (*P* < 0.0001).

#### 3.2.3 Central and peripheral fatigue

Table 2 shows the neuromuscular fatigue parameters for the two groups in the two conditions. Ptw decreased after exercise in both conditions (PL and ACT) in the T1D group (ACT: 138 ± 30 vs. 80 ± 18 N, *P *< 0.001; PL: 138 ± 16 vs. 84 ± 24 N, *P *< 0.001) and in the CTR group (ACT: 164 ± 14 vs. 75 ± 23 N, *P *< 0.001; PL: 167 ± 18 vs. 79 ± 16 N, *P *< 0.001). However, this reduction (∆Ptw) did not differ between the PL and ACT condition in the CTR group (−52 ± 12% vs. −54 ± 14%, respectively *P* = 0.74) and in the T1D group (−39 ± 13% vs. −41 ± 13%, respectively, *P* = 0.76) (Figure 2b). However, the magnitude of peripheral fatigue, quantified as the percentage reduction in Ptw from pre‐ to post‐exercise (ΔPtw), was greater in the CTR group than in the T1D group across conditions (*P* = 0.04). Similarly, MVC and VA decreased after exercise in both groups (*P* = 0.004), with no difference between PL and ACT condition (Figures 2a and 3c). Finally, there was no significant difference in *M*_max_ values between the two conditions in the two groups.

**FIGURE 2:**
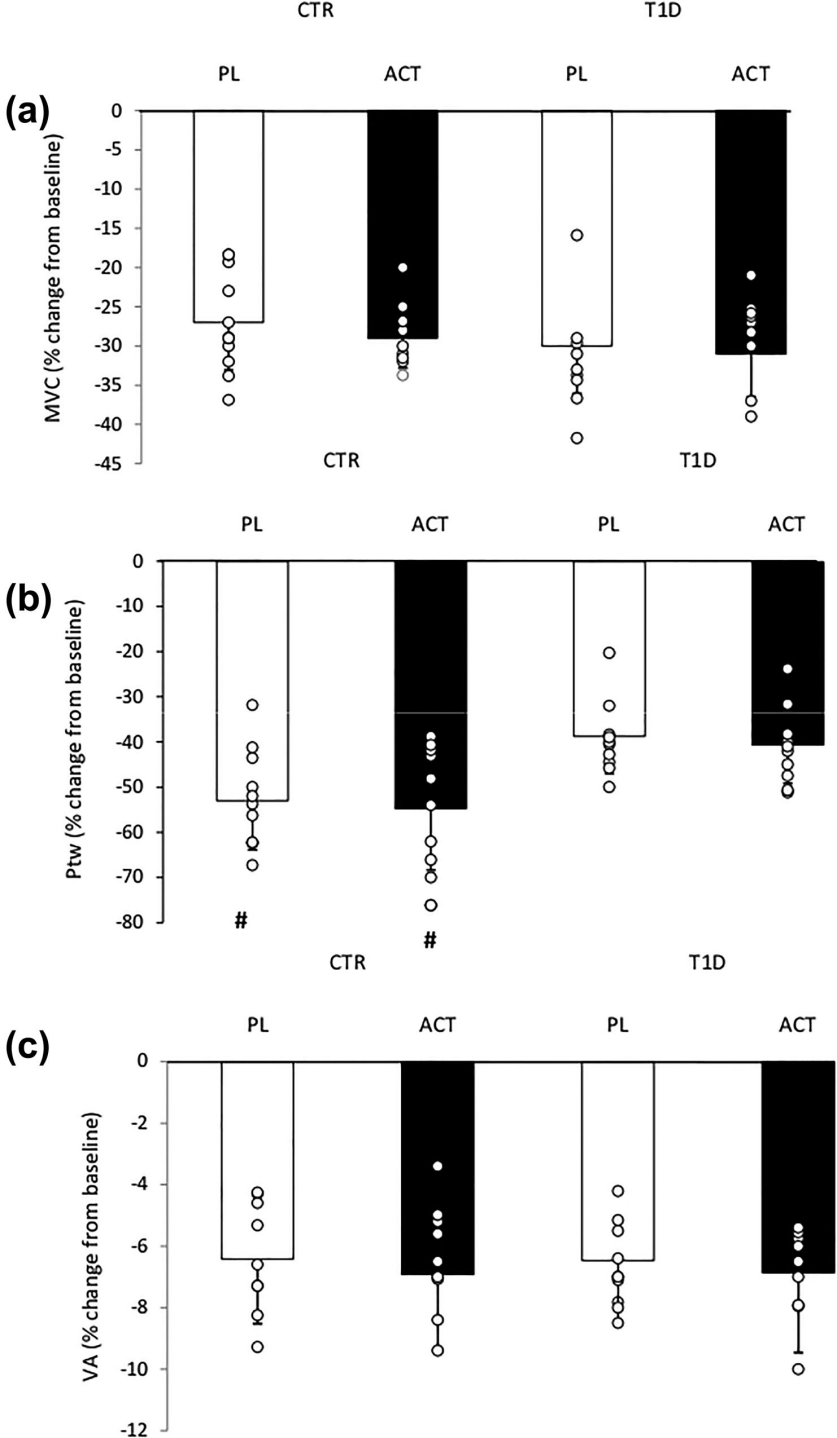
Neuromuscular fatigue parameters following exercise in control (CTR) and type 1 diabetes (T1D) participants. #*P *< 0.05 versus T1D. MVC, maximal voluntary contraction; Ptw, quadriceps potentiated twitch force; VA, voluntary quadriceps activation.

**FIGURE 3:**
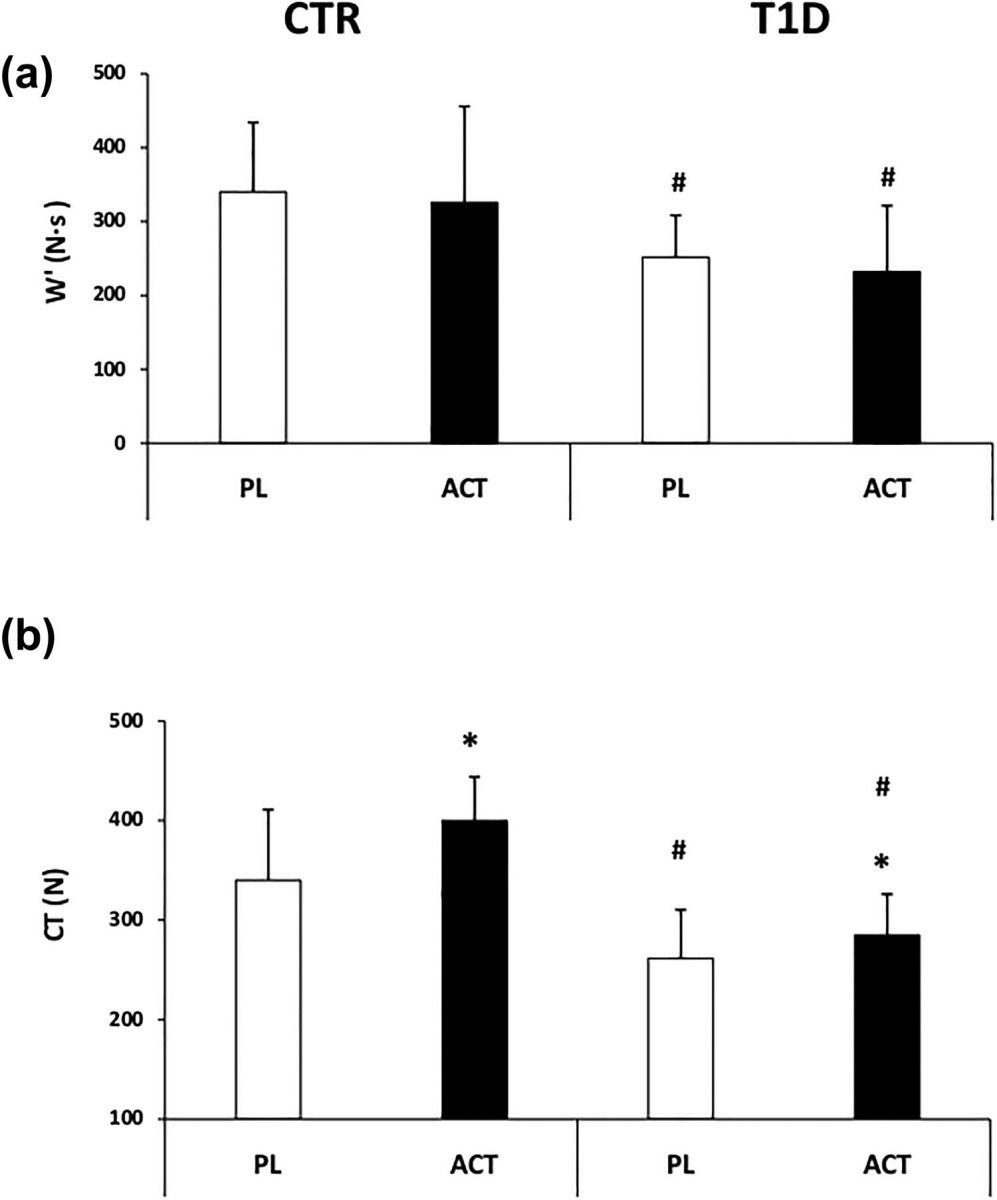
Amount of work performed (*W*′) (a) and critical torque (CT) (b) after exercise in PL and ACT conditions during exercise in CTR and T1D participants. ^∗^ *P* < 0.05 versus PL, #*P* < 0.05 versus CTR.

#### 3.2.4 Parameters associated with the force–duration relationship

Figure 3a shows that *W*′ values were lower in the T1D group compared with the CTR group in the PL and ACT conditions (*P* = 0.03). However, no significant difference was observed between the two conditions (PL vs. ACT) in either group (T1D: 252 ± 57 vs. 222 ± 90 N s, *P* = 0.41; CTR: 340 ± 94 vs. 326 ± 130 N s, *P* = 0.68, respectively).

Our results showed that CT values were lower in the T1D group compared with the CTR group in the PL and ACT condition (PL: T1D 262 ± 49 vs. CTR 340 ± 94 N; ACT: T1D 290 ± 81 vs. CTR 401 ± 71 N, *P* = 0.003). In addition, ACT increased CT values in both groups (*P* = 0.02) (Figure 3b). However, this improvement was significantly greater in the CTR group (+19 ± 3.4%) compared with the T1D group (+8.5% ± 2.2) (*P* = 0.001).

## 4 DISCUSSION

The present study examined the effects of ACT ingestion on force maintenance, muscle activation, CT, *W*′ and the magnitude of exercise‐induced peripheral fatigue quantified using ΔPtw, in T1D and matched healthy controls. In line with the hypotheses, ACT increased force output, CT, and RMS/*M*_max_ during the repeated‐MVC protocol in both groups, although the increases in CT and RMS/*M*_max_ were smaller in the T1D group. In contrast, ACT did not alter *W*′ or the magnitude of peripheral fatigue measured using ΔPtw.

In the present study, the development of fatigue and its underlying mechanisms were assessed during the performance of 60 MVC knee extension using the protocol described by Burnley et al. (2009). Our results showed no post‐exercise difference between ACT and PL conditions for the potential twitch (Ptw), voluntary activation level (VA) and M‐wave (*M*_max_) for the two groups. This observation aligns with Morgan et al. (2018), who reported that an attenuated development of neuromuscular fatigue was not accompanied by differential changes in central fatigue (voluntary activation), peripheral fatigue (potentiated twitch force, Ptw), or peripheral neuromuscular excitability (M‐wave) between ACT and PL trials in healthy participants. In the present study, although neuromuscular fatigue was still evident during the ACT condition, acute ACT ingestion increased MVC performance during the 60‐MVC protocol by ∼9% compared with PL in both groups. This finding is consistent with previous work showing that acute ACT can enhance performance during whole‐body exercise (Mauger et al., 2010; Foster et al., 2014).

EMG data showed an increase in RMS/*M*_max_ values during the 60‐MVC protocol in the ACT condition compared with the PL condition in both groups. Our results are in agreement with those reported by Morgan et al. (2018), who found an increase in RMS/*M*_max_ values with ACT ingestion compared to PL in healthy subjects. The authors suggested a possible effect of ACT ingestion on reducing III and IV afferent activity. During exercise, group III and IV ascending muscle afferents discharge in response to metabolic and mechanical stimuli in active skeletal muscle to regulate pain sensation, muscle activation and the development of peripheral fatigue (Pollak et al., 2014; Blain et al., 2016; Hureau et al., 2019). It is generally accepted that ACT primarily exerts its effects by inhibiting cyclooxygenase, the enzyme responsible for converting arachidonic acid into prostaglandins (Anderson, 2008). Because prostaglandins sensitize nociceptors (Rueff & Dray, 1993; Schaible et al., 2011), and cyclooxygenase inhibition can reduce group III/IV muscle afferent discharge during dynamic exercise (Hayes et al., 2006), these pathways may help explain the higher muscle activation (RMS/*M*_max_) observed during exercise following ACT ingestion in the present study.

CT is a parameter of the torque–duration relationship and, in the present protocol, represents the mean torque sustained during the final 12 contractions. CT should not be interpreted as a direct measure of a critical peripheral fatigue threshold. In contrast, ΔPtw quantifies the measured magnitude of exercise‐induced contractile impairment from pre‐ to post‐exercise. Therefore, the lower CT observed in the T1D group indicates a reduced capacity to sustain torque during repeated maximal contractions, whereas the between‐group difference in ΔPtw indicates a difference in the magnitude of peripheral fatigue developed during the task. These outcomes are conceptually distinct, and neither directly establishes the existence or level of a theoretical critical peripheral fatigue threshold.

Type 1 diabetes has been associated with a shift toward a greater proportion of type II fibres (Crowther et al., 2003; Fritzsche et al., 2008). Type II fibres typically display greater phosphocreatine stores (Karatzaferi et al., 2001), higher glycolytic enzyme content and ATPase activity, and lower oxidative capacity than type I fibres. During high‐intensity contractions, their elevated rates of ATP and phosphocreatine turnover promote the accumulation of fatigue‐related metabolites, including inorganic phosphate and ADP (Allen et al., 2008). Accordingly, during sustained isometric exercise, skeletal muscle in T1D may undergo greater metabolic disturbance, particularly in inorganic phosphate and hydrogen ion concentrations, which could contribute to the lower CT observed in this group. However, intramuscular metabolic changes were not assessed directly, and this interpretation remains speculative.

The increase in muscle activation in the ACT condition compared with the PLA condition was accompanied by a higher CT. This result is in line with those reported by Morgan et al. (2018) who observed a positive correlation between changes in EMG and critical torque with ACT administration. By reducing pain sensation, ACT ingestion could promote greater amplitude of intramuscular metabolic perturbation, leading to improved exercise tolerance (Mauger et al., 2010; Foster et al., 2014; Morgan et al., 2018). It is therefore possible that ACT ingestion reduced the development of fatigue by allowing greater muscle activation for a given degree of intramuscular metabolic perturbation. Furthermore, our results showed that the improvement in RMS/*M*_max_ values and CT during the 60‐MVC protocol with ACT was lower in the T1D group compared to the CTR group (RMS/*M*_max_: T1D +21% vs. CTR +40% ± 8%; CT: T1D +8%, CTR +19%). Thus, the smaller ACT‐related response observed in T1D may reflect differences in the physiological factors contributing to force regulation during exercise. However, because the underlying mechanisms were not directly assessed in the present study, this interpretation remains tentative. Although we did not directly alter afferent feedback in the present study, the specific mechanisms underlying the smaller ACT‐related response observed here remain unclear. Ishizawa et al. (2020) demonstrated that group IV muscle afferents in T1D rats have a greater response magnitude to mechanical stimuli (ramp‐shaped mechanical stimulation) than control animals (Ishizawa et al., 2020). Greater afferent responsiveness to a given metabolic or mechanical perturbation could potentially contribute to the smaller ACT‐related increases in muscle activation and CT in T1D. However, this interpretation remains tentative because group III/IV muscle‐afferent activity was not directly assessed. Moreover, given the tight relationship between intramuscular metabolic perturbation and peripheral fatigue (Broxterman et al., 2015; Blain, Mangum et al., 2016), differences in the excitability or central processing of group III/IV muscle‐afferent feedback may also have contributed to the smaller ACT response in T1D. Nevertheless, neither afferent feedback nor its central processing was measured directly. Further studies that specifically examine group III/IV muscle afferent feedback in individuals with T1D are needed to determine whether these mechanisms contribute to the blunted neuromuscular responses to ACT ingestion observed in the present study.

Another explanation could be that T1D patients exhibit high level of cyclooxygenase compared to CTR. Indeed, a recent paper has shown that cyclooxygenase is highly abundant in kidney tissue cells in a mouse model of type 1 diabetes (Nasrallah et al., 2003). Additionally, high levels of cyclooxygenase have been found in coronary arterioles obtained from diabetic patients (Szerafin et al., 2006). Therefore, as the effect of ACT is attributed to the inhibition of cyclooxygenase enzymes, it is thus possible that elevated level cyclooxygenase in T1D would blunt ACT ingestion effects on muscle activation and neuromuscular fatigue in the present study. Because group III/IV muscle afferent activity was not directly measured, any mechanistic interpretation involving afferent feedback should be considered speculative.

Some limitations inherent to the experimental protocol of this study warrant mention. First, having an accurate representation of the intracellular milieu (H^+^, Pi, Cr) may be indispensable to better understand neuromuscular fatigue mechanisms in T1D. Second, twitch interpolation is an efficient technique to explore the implication of central and peripheral factors in neuromuscular fatigue, and it would be interesting to explore the cortical activation using the transcranial magnetic stimulation in future investigations. Third, pain sensations were not assessed directly, as the protocol required participants to complete 60 MVCs with only brief recovery periods (2 s). Introducing pain ratings during this task could have distracted participants, compromised their focus, and potentially reduced their ability to sustain true maximal effort throughout the protocol. It is also important to note that this study was only performed on men and, given existing sex differences in neuromuscular fatigue development (Hunter, 2014), the conclusions may not be generalizable to women. Finally, the relatively small sample size should be considered when interpreting the between‐group difference in response magnitude, and larger studies are needed to confirm the generalizability of these findings.

### 4.1 Conclusion

Acute ACT ingestion improved force maintenance, CT and quadriceps muscle activation during repeated maximal contractions in both groups, although the increases in CT and RMS/*M*_max_ were smaller in patients with T1D. ACT did not alter *W*′ or the magnitude of peripheral fatigue quantified using ΔPtw. Thus, the ACT‐related increase in CT reflects an improvement in the capacity to sustain torque and should not be interpreted as evidence of a change in a critical peripheral fatigue threshold. These findings indicate a blunted neuromuscular response to ACT in participants with T1D. This smaller response may reflect differences in the physiological processes regulating muscle activation and force maintenance during repeated maximal contractions. However, because group III/IV muscle‐afferent activity, pain perception and intramuscular metabolic responses were not directly assessed, the mechanisms underlying this response remain uncertain.

Finally, combining ACT ingestion with neuromuscular assessment may therefore provide a hypothesis‐generating experimental approach for investigating neuromuscular responsiveness in T1D. However, the present findings do not support its use as a clinical detection or diagnostic method.

## AUTHOR CONTRIBUTIONS

Nadia Fekih, Amal Machfer, and Mohamed Amine Bouzid contributed to conceptualization. Nadia Fekih, Firas Zghal, and Mohamed Amine Bouzid contributed to methodology. Nadia Fekih, Amal Machfer, and Achraf Ammar contributed to formal analysis. Nadia Fekih and Firas Zghal contributed to investigation. Mohamed Amine Bouzid contributed to resources. Nadia Fekih contributed to data curation. Nadia Fekih and Amal Machfer contributed to writing—original draft preparation. Nadia Fekih, Firas Zghal, and Achraf Ammar contributed to writing—review and editing. Mohamed Amine Bouzid contributed to supervision. Nadia Fekih and Amal Machfer contributed to project administration. All authors have read and approved the final version of this manuscript and agree to be accountable for all aspects of the work in ensuring that questions related to the accuracy or integrity of any part of the work are appropriately investigated and resolved. All persons designated as authors qualify for authorship, and all those who qualify for authorship are listed.

## CONFLICT OF INTEREST

None declared.

## FUNDING INFORMATION

No funding was received for conducting this study.

## GENERATIVE AI STATEMENT

The authors confirm that no artificial intelligence tools, including large language models (LLMs), were used in the drafting or revision of this manuscript. All content was conceived, written and approved solely by the authors.

## Data Availability

The data may be shared upon reasonable request to the corresponding author if the request is accepted by the Regional Research Committee for Medical and Health Research Ethics and the local Data Protection Official.
